# A microscopic transungual surgical approach for subungual glomus tumor resection

**DOI:** 10.3389/fmed.2025.1607299

**Published:** 2025-08-14

**Authors:** Jianbo Zhong, Kamran Ali, Ping Yang, Xingyun Zhao, Inmaculada Xu Lou, Liming Wu

**Affiliations:** ^1^Department of Dermatology, Zhejiang Chinese Medical University, Zhejiang, China; ^2^Department of Dermatology, Affiliated Hangzhou First People's Hospital, School of Medicine, Westlake University, Zhejiang, China; ^3^Department of Surgery, The Fourth Affiliated Hospital of School of Medicine, International School of Medicine, International Institutes of Medicine, Zhejiang University, Zhejiang, China; ^4^Department of Cardiology, Hangzhou Hospital of Traditional Chinese Medicine, Zhejiang, China

**Keywords:** nail surgery, glomus tumor, subungual glomus tumors, transungual surgical approach, microscopic nail surgery

## Abstract

**Background:**

Glomus tumors, rare benign neoplasms developing from the glomus, often pose challenges in early detection due to their small size and subtle symptoms. Surgical removal remains the only primary treatment; however, existing approaches have limitations, particularly in preserving nail structure and minimizing recurrence rates. This study introduces the Microscopic Transungual Surgical Approach to overcome the drawbacks of existing procedures for glomus tumor excision and evaluate its efficacy in treating these tumors.

**Methods:**

We retrospectively analyzed 61 patients diagnosed with and treated for glomus tumors between 2014 and 2024, examining their clinical characteristics, diagnostic test results (Love’s and Hildreth’s tests), and MRI findings. The surgical approach included complete nail plate removal followed by microscopic tumor excision and attentive postoperative management.

**Results:**

The study included 61 glomus tumor patients [49 females (80.3%), 12 males (19.7%)] with a median age of 39 years. Tumors most commonly affected the thumb (39.3%) and ring finger (23.0%). Key clinical features included severe pain (98.4%), positive Love test (95.1%), and cold sensitivity (88.5%). Associated findings included nail deformities (50.8%) and bone changes (39.3%). At 1-year follow-up, 80.2% achieved satisfactory nail recovery, with a 4.9% recurrence rate.

**Conclusion:**

Our study suggests the potential efficacy of the Microscopic Transungual Surgical Approach in treating glomus tumors. Its enhanced visualization contributes to complete removal of tumors with less surrounding tissue damage to lower recurrence rates and improved patient outcomes.

## 1 Introduction

Glomus tumor is an uncommon benign neoplasm that develops in the glomus, a component of the dermis responsible for regulating temperature in the cutaneous microvasculature (1–3). This type of tumor typically manifests in the extremities, primarily in the fingers of the hands and feet (4), although it can also occur in other areas of the body (5–8). Normally, its size ranges around 1 cm in diameter, although cases of smaller size have also been reported (4, 9), which can be challenging to detect, especially in the early stages (3, 10). Consequently, delays in diagnosis are common (11). It is important to note that malignant transformation of glomus tumor is extremely rare, occurring in only 1% of cases (12, 13). However, careful clinical differentiation from subungual melanoma remains essential due to their potentially similar presentations (14, 15). Generally, glomus tumor affects approximately 75% of cases in the subungual region of the hands (16) and is more frequent in middle-aged women, aged between 30 and 70 years (3, 4, 17–19). The symptoms of glomus tumor include sensations of cold or heat in the affected finger (20, 21), accompanied by intense paroxysmal pain (17). Suspecting the presence of a glomus tumor is warranted if the patient experiences the characteristic triad of intense pain, extreme sensitivity especially against the change of environment temperature, and color changes of nail plate, particularly in the fingertips (11, 20, 21). The pain often becomes so intense that it affects the patient’s quality of life (9, 22), even impairing sleep (13). Additionally, as the tumor grows in size, it can deform the nail or bone (16). Additional diagnostic techniques comprise the Love test, Hildreth test (19), assessment of cold sensitivity, and employment of transillumination (4, 9, 16, 20). The Love test entails the utilization of a precise instrument to elicit pain in the affected area while observing the absence of pain in the surrounding unaffected regions. This method exhibits a sensitivity of 100% but low specificity. Conversely, the Hildreth test involves the application of a tourniquet to the affected limb and inflation to 250 mmHg. In a positive test, a reduction in pain over the lesion is expected upon tourniquet inflation, followed by a sudden rebound of pain in the lesion upon release of the tourniquet valve. The Hildreth test is estimated to possess a sensitivity of 92% and a specificity of 91% (13, 18). Imaging diagnosis comprises ultrasound, computed tomography, and magnetic resonance imaging (3, 23), although additional tests such as X-rays, angiography, and ultrasound can be performed to obtain a more detailed diagnosis (3). At present, surgical removal is the sole treatment option for glomus tumor (6, 13). However, its management is challenging due to the small surgical field, and attempts are often made to perform conservative excision in order to preserve the nail matrix (21). Nevertheless, current surgical approaches have inherent limitations (3, 16). In this study, we introduce the microscopic transungual surgical approach, which aims to mitigate these limitations and improve the treatment outcomes.

## 2 Materials and method

### 2.1 Patients and diagnosis

**Figure 1:**
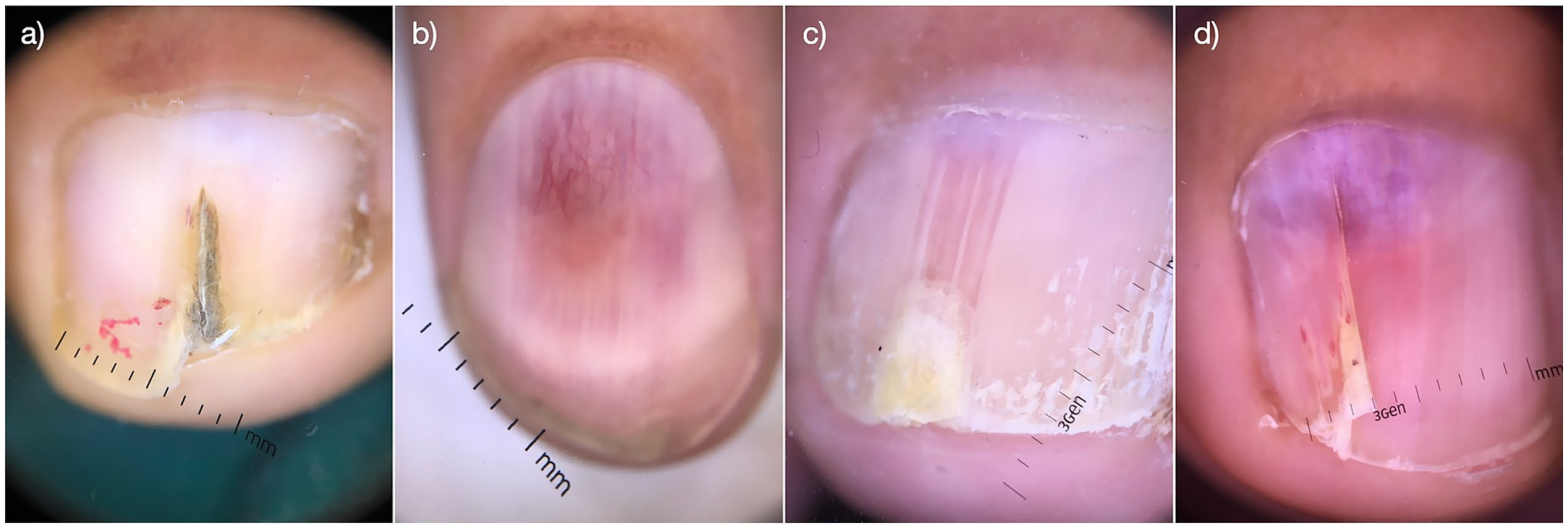
Dermoscopic images of the nail deformaties. Visual images of nail deformities in clinical cases before surgical removal **(a–d)**.

From 2014 to 2024, a total of 61 patients with glomus tumors were diagnosed and surgically treated at our hospital. The medical records of these patients were retrospectively reviewed, and the following data were extracted: sex, age, duration of the disease, follow-up time, presence of spontaneous severe pain, Love test results, Hildreth test results, cold stimulation, heat sensitivity, changes in the skin, bone changes, nail deformities, and affected fingers. MRI scans were performed on all patients to determine the size and location of the glomus tumor. Dermoscopy was used to evaluate the nail deformity (Figure 1). Nail recovery was evaluated during scheduled follow-up examinations at 6 months for fingernails and 12 months for toenails, with clinical assessment of regrowth parameters including plate morphology, texture, and adherence. Standardized diagnostic methods were employed to objectively document recovery progress and monitor potential recurrence. Written informed consent was obtained from all participants.

### 2.2 Imaging protocol and findings

**Figure 2:**
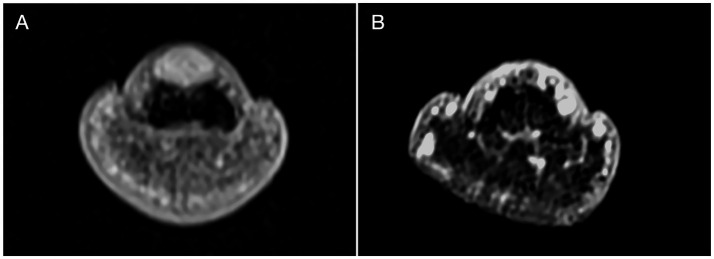
MRI imaging. **(A)** The T1W1 showed a well defined high signal dot sized 5x2x2mm was displayed in the subungual area. A depression was noticed on the dorsal part of distal phalanx corresponding to the site of high signal. Signal was more significant than T1W1 and relatively evenly distributed after enhancement. **(B)** MR investigation on 24 months follow up visit displayed no recurrence and noticeable recovery of the distal phalanx.

All patients underwent MRI evaluation using a Siemens Magnetom Verio 3.0 T scanner equipped with a 47 mm microscopic surface coil. The affected digit was carefully positioned within the coil, secured with medical tape, and stabilized using a sandbag to minimize motion artifacts. Standard imaging sequences included T1-weighted (T1WI), T2-weighted (T2WI), and post-contrast enhanced scans following gadolinium administration (24). MRI findings consistently demonstrated characteristic features of glomus tumors across all 61 cases. On T2WI, lesions exhibited uniformly high signal intensity, while T1WI revealed iso- to hypointense signals relative to muscle tissue (Figure 2). Post-contrast images showed marked homogeneous enhancement. Notably, 24 cases (39.3%) exhibited distal phalangeal depression, reflecting bony remodeling secondary to tumor compression (Table 1). These imaging characteristics provided reliable diagnostic confirmation and aided in preoperative planning.

**Table 1:** Clinical characteristics of patients.

	Frequency	Percentage
Gender		
Male	12	19.7
Female	49	80.3
Inadvertently severe pain		
Positive	60	98.4
Negative	1	1.6
Love test		
Positive	58	95.1
Negative	3	4.9
Cold stimulation experiments		
Positive	54	88.5
Negative	7	11.5
Hildreth test		
Positive	31	50.8
Negative	30	49.2
Heat sensitivity		
Positive	5	8.2
Negative	56	91.8
Skin changes		
Positive	11	18.0
Negative	50	82.0
Bone changes		
Positive	24	39.3
Negative	37	60.7
Nail deformity		
Positive	31	50.8
Negative	30	49.2
Affected fingers		
Thumb	24	39.3
Index finger	9	14.8
Middle finger	9	14.8
Ring finger	14	23.0
Little finger	5	8.2

**Figure 3:**
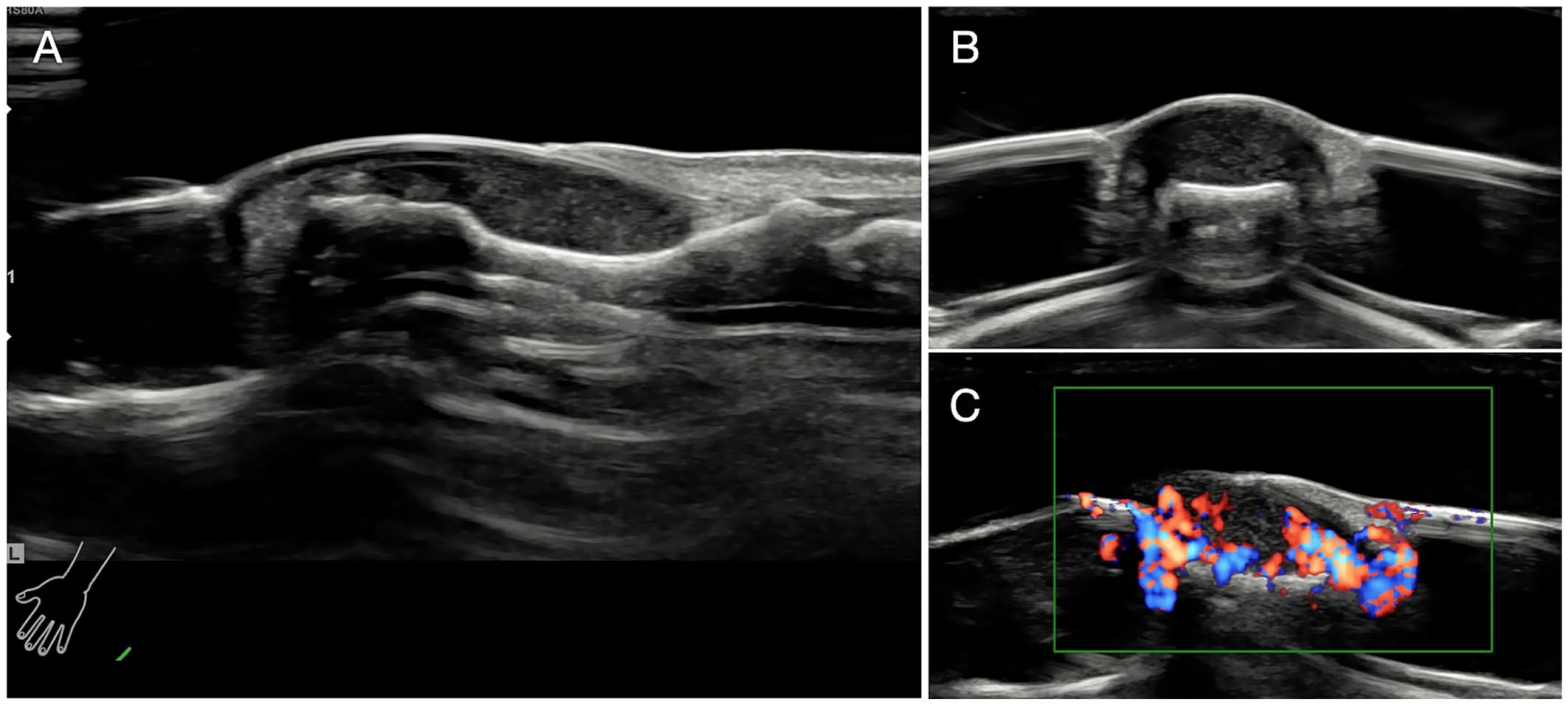
Ultrasonic investigation. **(A)** Glomus tumor exhibited regular shape and clear margin under ultrasonic examination. Tumors were low-echo without lateral shadows locating in the subungual area. **(B)** The ultrasonographic view of coronal section of the affected finger. **(C)** Abundant blood flow and vessels in and out of the tumors were observed.

Ultrasonic investigation were also taken to confirm the diagnosis and localization (Figure 3) (25, 26).

### 2.3 Surgical technique

**Figure 4:**
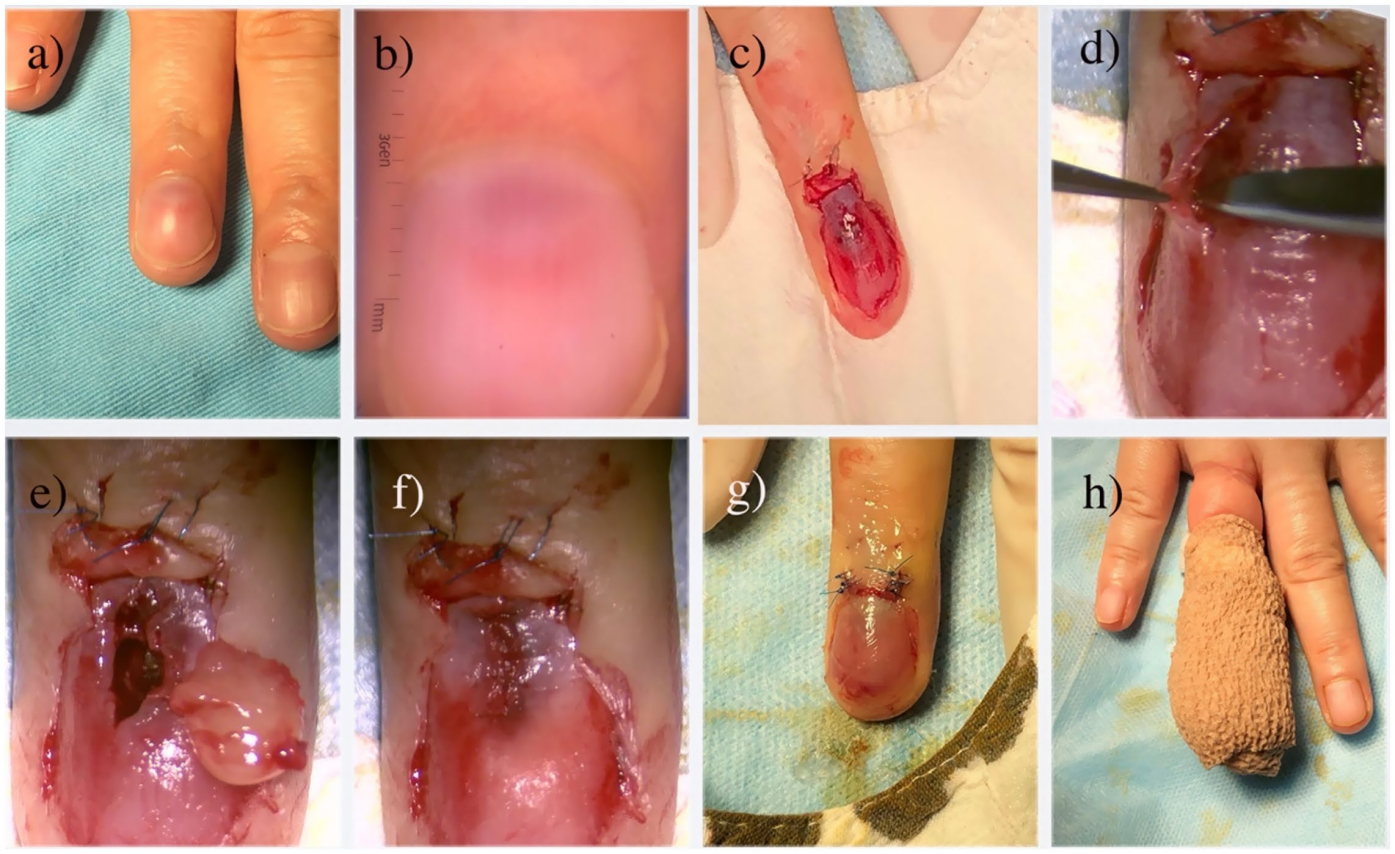
Microscopic transungual surgical approach for glomus tumor. **(a)** Gross appearance; **(b)** The glomus tumor is observed as a well-defined, homogeneous, red circular structure under dermoscopy; **(c)** Following the avulsion of the nail plate and lifting of the proximal nail fold, a view of the tumor body bearing a thin layer of the nail bed is revealed; **(d)** Careful dissection of the tumor undermicroscopy; **(e)** The completed resection of the tumor; **(f)** Closure of the nail bed using 8–0 absorbable sutures; **(g)** Repositioning of the proximal nail fold and replantation of the nail plate; **(h)** Application of a self-adhesive bandage as the final stage of the operation.

**Figure 5:**
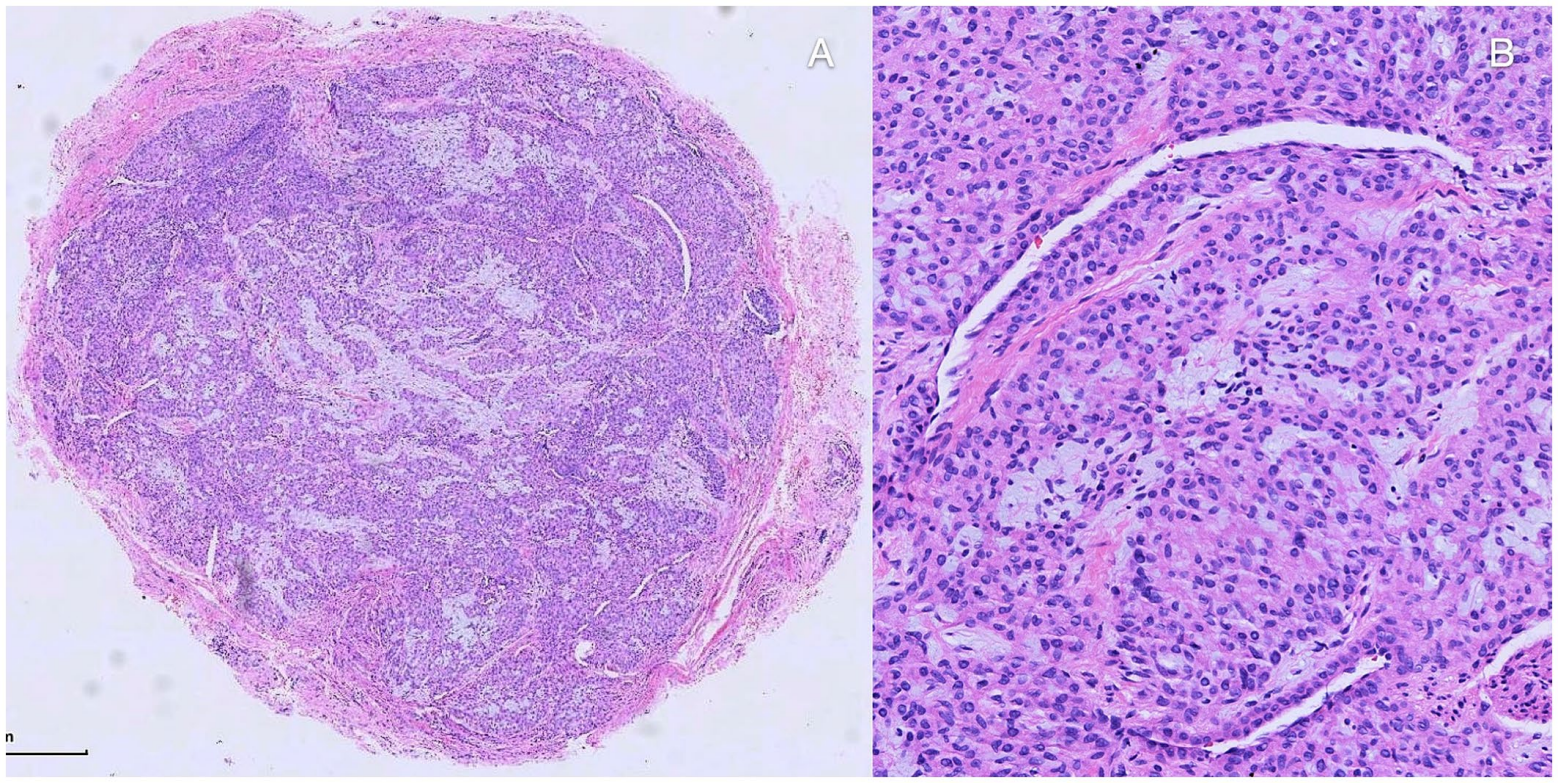
Histopathologic investigation. **(A,B)** Histopathologic investigation found well-defined nodule composed of nests of uniform, round to oval shape, glomus cells. It exhibited thin-walled, capillary-sized vessels surrounded by glomus cell nests and mature smooth muscle cells be noticed among the nests.

Patients were placed in a supine position, with the affected finger/toe immobilized, and standard disinfection were performed. A digital proximal block anesthesia was performed using 2% lidocaine until satisfactory numbness was achieved. The nail plate and nail bed were carefully separated using a dissecting instrument, and the entire nail plate was completely removed. The nail plate was thoroughly cleaned and stored in saline solution for later use. The proximal nail fold was vertically incised with tissue scissors, and the lifted proximal nail fold was secured to the dorsal skin of the finger/toe distal phalanx using 6–0 Prolene suture, exposing the entire nail bed and nail matrix (Figure 4c). Under a microscope, a No. 15 blade was used to make a midline longitudinal vertical incision along the elevated surface of the tumor, reaching the surface of the tumor(Figure 4d). The tumor was completely excised along its surface using micro scissors (Figure 4d), identifying the feeding and draining blood vessels, and electrocoagulation be performed precisely by micro-bipolar coagulation forceps in the connective tissue under the nail bed. If the tumor compressed the underlying bone surface, soft tissue was repeatedly scraped with a micro curette until the bone cortex was exposed, ensuring complete hemostasis. The wound was cleansed with PVP-I solution, and the nail bed/nail matrix was intermittently sutured with 8–0 Vicryl Coated suture, while the lifted proximal nail fold was repositioned using 6–0 Prolene suture (Figure 4f). The prepared nail plate was repositioned in its original position; the sutured area was covered with oiled gauze, followed by ointment and sterile dressing (Figure 4g). Finally, self-adhesive bandage was applied for hemostasis (Figure 4h). All specimen was immediately sent for histopathological confirmation to verify complete removal and establish definitive diagnosis (Figure 5).

### 2.4 Statistical analysis

All data were analyzed using SPSS version 21.0. For quantitative variables, the mean and standard deviation were reported when the data followed a normal distribution; otherwise, the median and interquartile range (Q1–Q3) were used. For qualitative variables, absolute and relative frequencies were calculated and expressed as percentages.

## 3 Results

A total of 61 patients with glomus tumors were included in the study, comprising 49 females (80.3%) and 12 males (19.7%). The median age of the overall sample was 39 years. Median age was 37 years for males and 39 years for females. The median disease duration was 5 years, with an interquartile range (Q1–Q3) of 3 to 10 years (Table 2). Regarding the location of the glomus tumor, we found that 24 (39.3%) were located in the thumb, 9 (14.8%) in the index finger, 9 (14.8%) in the middle finger, 14 (23.0%) in the ring finger, and 5 (8.2%) in the little finger. In terms of the clinical characteristics of glomus tumors, we found that 60 (98.4%) of the patients experienced inadvertently severe pain, 58 (95.1%) of the patients tested positive on the Love test, 31 (50.8%) tested positive on the Hildreth test, 54 (88.5%) tested positive on cold stimulation experiments, and 5 (8.2%) tested positive for heat sensitivity. Furthermore, regarding comorbidities associated with glomus tumors, it was found that 31 (50.8%) presented nail deformities, 24 (39.3%) showed bone changes, and 11 (18.0%) had skin changes. Follow-up was conducted for all patients, 49 (80.2%) of them reported a satisfying recovery of the nail plate. And 3 (4.9%) of them experienced recurrences. More detailed data are included in Tables 1, 2.

**Table 2:** Clinical characteristics of patients.

	Mean	SD	Minimum	Maximum
Follow-up time (until May 2024), months				
Total	31.61	7.26	20	48
Male	28.92	5.78	20	37
Female	32.27	7.48	20	48

	Median	Q1	Q3
Age, years			
Total	39	32	48
Male	37	30	52
Female	39	32	47
Disease duration, years			
Total	5	3	10
Male	4	2,25	17,50
Female	5	3	10

## 4 Discussion

The glomus tumor typically presents as a solitary entity (3) and is commonly located in the central proximal region (19). The transungual method provides a wide surgical field of vision and the benefit of accurate exposure to the tumor situated in the central area. However, on account of the limited accommodation of the subungual tissue, general surgical procedure can lead to postoperative nail deformities due to damage to the nail bed, resulting in a high incidence of nail deformities following the procedure (2). Inadequate surgical technique can cause nail deformities and increase the risk of tumor recurrence (4). The transungual approach improves tumor exposure and facilitates complete removal, thus reducing the risk of local recurrence (4, 18). However, this procedure entails complete nail extraction to access the glomus tumor, which can have negative cosmetic repercussions and severely damage the nail bed. Furthermore, if nail bed sutures are not performed carefully, they may cause nail deformities after surgery (3). Complete excision of the nail is the most effective method for full access to the tumor but can result in cosmetic issues and require the entire nail to regrow, leading to patient discomfort (18, 20). In the transungual approach, the removal of the nail plate is performed first, followed by a longitudinal incision in the nail bed and/or the nail matrix to extract the tumor. While this approach provides good visualization and precise tumor localization, it can also induce iatrogenic nail deformities ranging from a longitudinal ridge to complete nail division (27).

The lateral subperiosteal approach is preferable for glomus tumors located in the lateral zone. It is not effective in exposing centrally located tumors (2, 4). Among its advantages is the reduced risk of nail deformity (27), but it is limited by the nail’s location, making the procedure more complex. Occasionally, it can cause nerve damage (3, 16, 20). This type of surgery does not involve nail removal; however, it can sometimes lead to finger or fingertip deformities. The occurrence of these issues can be minimized depending on the glomus tumor’s location and the surgeon’s expertise (20). In some cases, this surgery may leave a tender sensation in the scar area, although it usually improves within 3–4 weeks (20). The surgical field is smaller than in the transungual approach, therefore, it reduces the risk of nail deformity. However, if the excision of the glomus tumor is incomplete, there is a higher risk of recurrence (27). In the literature, other approaches are also described, such as the modified periungual approach method. It involves making an L-shaped excision over the periungual area to the mid-lateral line. However, this method appears to be suitable only for tumors located in the marginal subungual region and requires a significant amount of time and skilled surgical techniques. Additionally, postoperative nail deformity may be more frequent than expected (27). Upon tumor removal, symptoms are alleviated or disappear (18), and typically there are no recurrences or complications (3). Tumor recurrences often occur if the tumor excision has not been complete (2). Therefore, it is necessary to remove the entire tumor in one procedure, with careful attention to detail (1, 3, 20). According to Kim et al., there does not appear to be a statistically significant difference in tumor recurrence based on the type of surgery (2). However, this is still a topic of debate due to the limited number of studies available on this subject. An innovative and current technique for addressing subungual glomus tumor is microscopic excision. This technique is considered superior in terms of recurrence prevention (16). Its advantages include preservation of finger function, nail esthetics, and increased patient satisfaction (16). Lambertini M reported that Mohs micrographic surgery can reduce the risk of recurrence, and annoying symptomatology. More researches are required for the conclusion of the outcome compared to other techniques (28). Minimally invasive transungual technique provides a small window for excision of digital glomus tumor. This technique brings benefits of fast recovery and satisfactory from patients for the minimal trauma of the nail unit (29). However, the exposure is limited, especially under the circumstance of large size tumor. By our procedure, tumors are excised precisely with assistant of microscope. And the careful avulsion and reposition of the intact nail plates bring satisfying recovery and cosmetic outcome by our result. An incision is made based on the tumor’s anatomical location (4). Additionally, performing an incision according to the anatomical location, providing appropriate anesthesia, and using a pneumatic tourniquet enhance tumor visualization and facilitate its removal, reducing the risk of postoperative complications (16). Meticulous repair of the nail bed after tumor excision reduces the risk of nail deformities (18). Careful dissection with the use of a microscope is helpful in completely eliminating the tumor and distinguishing lesions in the pulp from the surrounding tissue during surgery, providing a more precise tumor delineation. Moreover, this approach allows for meticulous handling and repair of the nail bed tissues (2). Bae et al. suggest in their study that the eponychial flap elevation method could be used as it may minimize nail dystrophy, minimize nail bed damage, as well as postoperative damage (30). However, we have only found one article on the use of this technique in glomus tumors, so the information is limited. Although there are no studies on this matter, we suggest that combining this approach with a microscope could enhance the effectiveness of this procedure. MRI is an excellent imaging modality for diagnosing glomus tumors minimum up to 2 mm in size. Typically, a MRI shows the glomus tumor as a central high-signal point surrounded by an area of lower signal intensity (20, 31). MRI can be useful in distinguishing glomus tumors from neuromas, melanomas, hemangiomas, and other foreign bodies (20, 32). It is the most accurate and detailed method (18), but it is costly. Ultrasound is less expensive but also less specific. Ultrasound may significantly underestimate the diameter of a glomus tumor. X-rays are inexpensive but do not reveal much unless there is bone involvement (21). The classic presentation of a glomus tumor is a subungual or periungual nodule of the distal phalanx. Recurrences can occur if satellite lesions were not identified during surgery. These satellite lesions can grow and become symptomatic, requiring further surgical intervention (1). MRI can be used to identify hidden and multiple glomus tumors (1). Although glomus tumors infiltrating the bone are rare, when this occurs, bone removal is also necessary. MRI is recommended if the bone is affected. In such cases, preoperative plain radiographs may also be considered (12). Studies such as Lin et al. report that factors such as changes in skin color in the area where the tumor is located can increase the risk of recurrence. This is because it becomes more challenging to delineate the affected area when there are changes in the skin. The more accurately the glomus tumor area is determined, the lower the chances of recurrence (21).

Among the main advantages of our study, several distinctive characteristics stand out. Firstly, we have included a significantly larger number of patients compared to previous studies (3, 16, 20). All patients in our study were diagnosed and treated following a uniform methodology. Additionally, we conducted both the Love test and the Hildreth test on all patients. All our patients received an intervention based on the Microscopic Transungual Surgical Approach technique. This treatment homogeneity provides us with the advantage of being able to compare results for future investigations. This study describes the surgical technique in detail, with particular attention to the microscopic transungual approach, which has been relatively underreported in the literature. While all included cases were histopathologically confirmed as glomus tumors, not all exhibited the classic clinical trial or uniformly positive diagnostic test results. These findings suggest that magnetic resonance imaging (MRI) serves as a valuable diagnostic tool when combined with clinical investigations. Regarding gender differences, other studies have revealed that, while there are variations in frequencies, a constant remains: the number of women affected by this disease is greater than the number of men (33).

This study has several limitations, including its retrospective design, partial dataset availability, and relatively small sample size, as well lake of incorporating validated patient-reported outcome. Additionally, the absence of a control or comparison group limits the generalizability of the findings (Table 3).

**Table 3:** Nail surgical procedures for glomus tumor.

Surgical technique	Pros	Cons
Transungual approach (29, 34, 35)	- Direct visualization of tumor - Effective for central and proximal lesions	- Risk of nail deformity - Postoperative pain - Recurrence possible
Transungual w/Nail preservation (29)	- Minimizes nail deformity - Rapid healing	- May be technically demanding for larger tumors - Recurrence possible
Sub-nail bed (Nail-sparing) approach (2, 36)	- Preserves nail plate - Reduces nail deformity - Good cosmetic outcomes - Effective for distal lesions	- Limited by tumor location - May not suit very large/complex tumors - Insufficient exposure of leisons
Lateral subperiosteal approach (2, 37)	- Good for lateral lesions - Preserves most of nail bed	- Lesser exposure for centrally located tumors - Technical challenge - Difficulty in maintaining position of ulnar side lesions of most digits.
Modified nail folding approach (38)	- Nail preservation - Excellent visualization - Fast recovery	- Technique-dependent - Still under evaluation - Time consuming
Mohs micrographic surgery (28, 39–41)	- High cure rates - Maximum tissue conservation	- Time-consuming - Costly - Requires expertise and stages - Still under evaluation

## 5 Conclusion

The Microscopic Transungual Surgical Approach could be a promising option for treating glomus tumors. This technique has the potential to minimize the disadvantages associated with other surgical approaches. Further studies are needed to investigate the effectiveness of this surgical technique.

## What is known on this topic?

Glomus tumors are rare benign neoplasms commonly found in the subungual region of the hands, often causing intense pain and nail deformities. Surgical removal is the only effective treatment, but existing approaches have limitations such as incomplete tumor excision, nail deformities, and recurrence.

## What does this study add?

This study evaluates the effectiveness of the Microscopic Transungual Surgical Approach, demonstrating its potential to achieve complete tumor removal while reducing recurrence rates and nail deformities compared to conventional surgical methods.

## How might this study affect research, practice, or policy?

The findings could influence surgical practice by promoting the adoption of microscopic transungual surgery as a preferred technique for glomus tumor excision, potentially improving patient outcomes and reducing recurrence rates in clinical settings.

## Data Availability

The original contributions presented in the study are included in the article/supplementary material, further inquiries can be directed to the corresponding author/s.
